# The role of SARC-F scale in predicting progression risk of COVID-19 in elderly patients: a prospective cohort study in Wuhan

**DOI:** 10.1186/s12877-021-02310-x

**Published:** 2021-06-10

**Authors:** Yao Ma, Min He, Li-Sha Hou, Shen Xu, Zhi-Xin Huang, Na Zhao, Yan Kang, Ji-Rong Yue, Chenkai Wu

**Affiliations:** 1 Department of Geriatrics and National Clinical Research Center for Geriatrics, West China Hospital, Sichuan University, Chengdu, Sichuan Province China; 2 COVID-19 Medical Assistance Teams (Hubei) of West China Hospital, Sichuan University, Chengdu, Sichuan Province China; 3 Department of Critical Care Medicine, West China Hospital, Sichuan University, Chengdu, Sichuan Province China; 4 Department of Endocrinology and Metabolism, West China Hospital, Sichuan University, Chengdu, Sichuan Province China; 5 Department of Obstetrics and Gynecology, Renmin Hospital of Wuhan University, Wuhan, Hubei Province China; 6 Department of Otolaryngology-Head and Neck Surgery, Renmin Hospital of Wuhan University, Wuhan, Hubei Province China; 7 Global Health Research Center, Duke Kunshan University, Suzhou, Jiangsu Province China

**Keywords:** COVID-19, SARC-F, Sarcopenia, Prognosis, Older

## Abstract

**Background:**

Since the outbreak of COVID-19, it has been documented that old age and underlying illnesses are associated with poor prognosis among COVID-19 patients. However, it is unknown whether sarcopenia, a common geriatric syndrome, is associated with poor prognosis among older COVID-19 patients. The aim of our prospective cohort study is to investigate the association between sarcopenia risk and severe disease among COVID-19 patients aged ≥60 years.

**Method:**

A prospective cohort study of 114 hospitalized older patients (≥60 years) with confirmed COVID-19 pneumonia between 7 February, 2020 and 6 April, 2020. Epidemiological, socio-demographic, clinical and laboratory data on admission and outcome data were extracted from electronic medical records. All patients were assessed for sarcopenia on admission using the SARC-F scale and the outcome was the development of the severe disease within 60 days. We used the Cox proportional hazards model to identify the association between sarcopenia and progression of disease defined as severe cases in a total of 2908 person-days.

**Result:**

Of 114 patients (mean age 69.52 ± 7.25 years, 50% woman), 38 (33%) had a high risk of sarcopenia while 76 (67%) did not. We found that 43 (38%) patients progressed to severe cases. COVID-19 patients with higher risk sarcopenia were more likely to develop severe disease than those without (68% versus 22%, *p < 0.001*). After adjustment for demographic and clinical factors, higher risk sarcopenia was associated with a higher hazard of severe condition [hazard ratio = 2.87 (95% CI, 1.33–6.16)].

**Conclusion:**

We found that COVID-19 patients with higher sarcopenia risk were more likely to develop severe condition. A clinician-friendly assessment of sarcopenia could help in early warning of older patients at high-risk with severe COVID-19 pneumonia.

## Background

The coronavirus disease 2019 (COVID-19) is an acute respiratory infectious disease caused by infection with SARS-CoV-2 (Severe acute respiratory syndrome coronavirus 2). COVID-19 is highly contagious and could lead to a high rate of severe illness [1, 2]. Since the outbreak in December 2019, COVID-19 has swept the world and more than two hundred countries and regions have been affected. As of 13 April, 2021, there have been 136,115,434 confirmed cases of COVID-19, including 2,936,916 deaths [3].

About 15% of COVID-19 cases experienced severe disease (covid-strategy-update-14april2020.pdf) and the fatality rate ranged from 1.4 to 14% among hospitalized patients [4–6]. Studies have shown that COVID-19 patients with older age, comorbidities, lactic dehydrogenase (LHD) and D-dimer, are at more likely to develop severe complications and die [7–9]. Older adults are more vulnerable to be infected and at higher risk of developing serious complications with COVID-19. The mortality rate for older people could be incredibly high and meanwhile the fatality rate of those over 80 years old is five times the global average (covid-strategy-update-14april2020.pdf). The COVID-19 pandemic has posed a disproportionately high threat to older adults [10–12]. Older adults represent a diverse population with hugely different health status; chronological age alone may not capture the full risk spectrum and may not truly reflect the differences underlying the biological ageing process. Therefore, it is scientifically unreasonable to give up treatment based on chorological age alone.

The concept of sarcopenia was originally proposed by Rosenberg in 1989. Sarcopenia is characterized by a progressive decline in skeletal muscle mass, power and strength, whereas frailty still requires the loss of performance [13, 14]. The prevalence of sarcopenia is about 5 to 70%, depending on age, sex and different diagnostic criteria [15–18]. Sarcopenia is strongly associated with falls, fractures, disability, and high healthcare expenditures [19]. Studies have shown that frailty was a better predictor for prognosis of COVID-19 than either age or comorbidity [20, 21]. Sarcopenia shares the similar pathogenesis with frailty and is often considered as a precursor to frailty [22, 23]. We assume that age-related sarcopenia is a risk factor for the aggravation of the patient’s condition. Early identification of patients with a high risk of sarcopenia can help us advance the timing of intervention to avoid the deterioration of the patient’s condition to an extremely serious state and a state requiring a lot of resources. We conducted a prospective cohort study to investigate the association between sarcopenia, and progression risk among older COVID-19 patients. Sarcopenia was assessed the SARC-F scale which is an inexpensive and clinic-friendly screening tool with satisfactory reliability and specificity [24].

## Methods

### Study design and participants

This is a prospective cohort study conducted in East Campus of Renmin Hospital of Wuhan University/Hubei General Hospital by COVID-19 medical team (Hubei) of West-China hospital. This study was performed according to the Helsinki Declaration and approved by the Ethics Committee of West China Hospital. Because no paper documents are allowed to be taken out of the quarantine area, the requirement for written informed consent was waived by the Ethics Committee of West China Hospital and informed consent was obtained verbally (Y.M) from all participants.

A total of 114 patients over 60 years old newly diagnosed with COVID-19 from February 7 to April 6, 2020 were enrolled in this study. The inclusion criteria were: (1) age ≥ 60 years; (2) diagnosis of COVID-19 pneumonia confirmed by positive result for COVID-19 RNA in nasopharyngeal swabs according to the WHO interim guidance published on 28 January, 2020 [25]; (3) completion of SARC-F assessment on admission; (4) availability of relevant medical record information. Patients with the following conditions will be excluded: incomplete information; don’t comply with treatment; don’t comply with questionnaire survey; Mental disorders and consciousness disorder; be critically ill when admitted.

### Data and specimen collection

#### Sarcopenia risk

We evaluated patients through face-to-face interviews and completed the SARC-F scale by experienced geriatricians within 24 h of admission. We used the SARC-F scale to measure risk of sarcopenia. Five components were included: strength; assistance walking; rise from a chair; climb stairs; and falls. Each type of evaluation item contains 0 points, 1 point, and 2 points according to the degree of difficulty or frequency. The total score ranges from 0 to 10. Patients with a total score ≥ 4 were considered to have a high risk of sarcopenia [24].

#### Socio-demographics and clinical conditions

Patients’ demographics (age and sex), health behaviors (smoking and body mass index [BMI]), medical history, comorbidities (hypertension, diabetes, coronary heart disease, stroke, cancer, chronic pulmonary disease, and chronic kidney disease), vital sign on admission, clinical conditions (fever, fatigue, myalgia, pharyngalgia, dry cough, expectoration, hemoptysis, dyspnea, chest pain, anorexia, diarrhea, nausea) and laboratory data (white blood cell count, lymphocyte count, haemoglobin, albumin, creatinine, CD8+, D-dimer, C-reactive protein [CRP]) were assessed within 24 h of admission at baseline. Subsequently, changes in the condition (improvement or progression of disease) were evaluated until hospitalization day 60 or death/discharge.

#### Laboratory data

Blood samples were taken within 24 h of hospital admission . Laboratory data (white blood cell count, neutrophil count, lymphocyte count, hemoglobin, albumin, creatinine, CD8+, D-dimer, and C-reactive protein [CRP]) on admission were assessed. We managed the patients in accordance with WHO’s guideline and official guideline of China, and closely monitored patients’ condition changes [26, 27].

### Outcomes

The outcome in our study was development of severe disease within 60 days of hospital admission, defined as fever or suspected respiratory infection plus one of the following conditions according to the WHO Interim guidance for COVID-19: respiratory rate > 30 breaths/min; severe respiratory stress; or SpO2 ≤ 93% on room air, as well as acute respiratory distress syndrome (ARDS) which is diagnosed according to the Berlin criteria [27, 28]. Individuals were censored when they were discharged or the end of the analytic period (60 days), whichever came first. Follow-up information was available through April 6, 2020.

### Statistical analysis

We used SAS 9.4 (SAS Institute Inc., Cary, NC, USA) to perform data analysis. We evaluated the normality of the distribution of variables using the Kolmogorov–Smirnov test. Normally distributed data were expressed as mean ± SD while non-normally distributed data were expressed as median (interquartile range). The difference between patients with and without sarcopenia was tested by independent Student’s t-test for normally distributed variables and Mann–Whitney U test for non-normally distributed variables. The chi-square test or Fisher exact test was used to compare categorical variables between patients with and without sarcopenia.

We first calculated the incidence rate of severe disease among the overall sample and by sarcopenia status. We then used the Kaplan–Meier method to plot survival curves. Differences were examined using the log-rank test. We used the Cox proportional hazards models to identify the unadjusted and adjusted associations between sarcopenia and severe disease. All tests were two-sided and *P* values of ≤0.05 were considered as being statistically significant.

## Results

### Baseline characteristics

In this prospective cohort study, a total of 114 patients were included (Table 1). The mean age was 69.52 ± 7.25 years, and 57 (50%) were women. The average age among patients with low risk of sarcopenia was 68.93 ± 7.00 years and was 72.05 ± 8.36 years among patients with a high risk of sarcopenia (*p* = 0.038). There were no statistically significant differences in comorbidities between two groups. There were proportionally more individuals feeling fatigue (74% versus 50.00%, *p* = 0.019), dyspnea (58% versus 32%, *p* = 0.007) and anorexia (74% versus 54%, *p =* 0.042) in patients with a high risk of sarcopenia than those with lower risk. Patients with a high risk of sarcopenia have statistically significantly higher level in white blood cell, neutrophil, hemoglobin, D-dimer, and procalcitonin than those with lower risk. The lymphocyte count, CD3+, CD4+, and CD8+ were statistically significantly lower among patients with a high risk sarcopenia than those with lower risk.

**Table 1:** Demographic, clinical, laboratory findings of patients on admission

Characteristics	Total(***n*** = 114)	Low risk of Sarcopenia (***n*** = 76)	High risk of sarcopenia (***n*** = 38)	***p*** value
Age, Years	69.52 ± 7.25	68.93 ± 7.00	72.05 ± 8.36	0.038
**Sex**				
Female	57(50.00%)	38(50.00%)	19(50.00%)	1.000
Male	57(50.00%)	38(50.00%)	19(50.00%)	
Body Mass Index	23.46 ± 3.18	23.57 ± 3.23	23.20 ± 3.08	0.576
**Smoking**	19(16.67%)	12(15.79%)	7(18.42%)	0.722
**Comorbidity**				
Hypertension	57(50.00%)	37(48.68%)	20(52.63%)	0.691
Diabetes	20(17.54%)	14(18.42%)	6(15.79%)	0.728
Coronary heart disease	10(8.77%)	4(5.26%)	6(15.79%)	0.061
Stroke	6(5.26%)	2(2.63%)	4(10.53%)	0.075
Cancer	9(7.89%)	7(9.21%)	2(5.26%)	0.461
Chronic pulmonary disease	14(12.28%)	8(10.52%)	6(15.79%)	0.420
Chronic kidney disease	7(6.14%)	4(5.26%)	3(7.89%)	0.581
**Symptoms**				
Fever	87(76.32%)	60(78.95%)	27(71.05%)	0.350
Fatigue	66(52.63%)	38(50.00%)	28(73.68%)	0.019
Myalgia	10(8.77%)	6(7.89%)	4(10.53%)	0.640
Pharyngalgia	4(3.51%)	3(3.95%)	1(2.63%)	0.710
Dry cough	72(63.16%)	47(61.84%)	25(65.79%)	0.680
Expectoration	37(32.46%)	24(31.58%)	13(34.21%)	0.777
Hemoptysis	5(4.39%)	4(5.26%)	1(2.63%)	0.518
Dyspnea	46(40.35%)	24(31.58%)	22(57.89%)	0.007
Chest pain	4(3.51%)	2(2.63%)	2(5.26%)	0.472
Anorexia	69(60.53%)	41(53.95%)	28(73.68%)	0.042
Diarrhea	12(10.53%)	9(11.84%)	3(7.89%)	0.517
Nausea	11(9.65%)	6(7.89%)	5(13.16%)	0.370
**Laboratory findings**				
White blood cell count (× 10^9^/L)	6.50(5.28–8.52)	6.48(5.34–8.51)	6.85(4.89–9.72)	0.016
Neutrophil count (×10^9^/L)	4.19(3.01–5.91)	4.78(3.40–5.95)	5.14(3.46–8.59)	0.002
Lymphocyte count (×10^9^/L)	0.82(0.58–1.13)	0.90(0.66–1.35)	0.69(0.52–1.12)	0.002
Platelet (×10^9^/L)	204.00(161.00–265.00)	199.00(171.00–261.00)	215.50(152.00–270.50)	0.766
Hemoglobin, g/L	120.00 ± 20.91	116.87 ± 21.06	126.50 ± 18.46	0.018
Albumin, g/L	34.90(32.10–37.45)	34.10(32.20–36.50)	36.85(32.30–38.25)	0.147
Creatinine, μmol/L	65.00(53.00–79.00)	61.00(53.00–82.00)	65.00(49.00–73.00)	0.274
D-Dimer, mg/L	1.55(0.69–4.65)	1.07(0.56–2.47)	2.23(1.17–7.80)	0.013
C-reactive protein, mg/L	59.10(21.30–110.20)	61.30(21.30–110.70)	53.05(21.75–99.00)	0.651
Procalcitonin, ng/mL	0.09(0.06–0.21)	0.07(0.05–0.22)	0.10(0.06–0.18)	0.003
BNP, pg/mL	270.50(135.00–661.00)	266.80(122.70–457.50)	348.40(159.50–813.70)	0.027
CD + 3, count/μl	518.00(317.00–804.00)	538.00(345.00–850.00)	432.00(235.00–707.00)	0.007
CD + 4, count/μl	348.00(212.00–513.00)	378.00(230.00–559.00)	277.00(150.50–476.50)	0.006
CD + 8, count/μl	167.00(81.00–279.00)	193.00(94.00–258.00)	122.50(76.00–285.00)	0.013

Data are median (IQR), n (%). *p* values were calculated by Mann-Whitney U test, χ^2^ test, or Fisher’s exact test, as appropriate

*Abbreviations*: *BNP* Brain Natriuretic Peptide

### Primary outcome: differences between high risk and low risk of sarcopenia

Forty-three of 114 (38%) patients progressed to severe disease including eight deaths during the follow-up period, and the overall incidence rate of severe disease was 1.48 per 100 person-days (95% confidence interval [CI]: 1.10–1.99; Table 2). We found 22% (17/76) patients with low risk and 68% (26/38) patients with high risk became severe. The incidence rate of severe disease among patients with high and low risk of sarcopenia was 0.77 per 100 person-days (95% CI: 0.48–1.24) and 4.41 per 100 person-days (95% CI: 3.01–6.48), respectively.

**Table 2:** Overall incidence rate of severe illness and incidence rate by Sarcopenia risk

Sarcopenia risk	Number of severe diseases	Total person-days	events per 100 person-days (95% CI)
Total (n = 114)	43	2908	1.48(1.10–1.99)
Low risk (n = 76)	17	2198	0.77(0.48–1.24)
High risk (n = 38)	26	589	4.41(3.01–6.48)

*Abbreviations*: *CI* Confidence interval

**Fig. 1:**
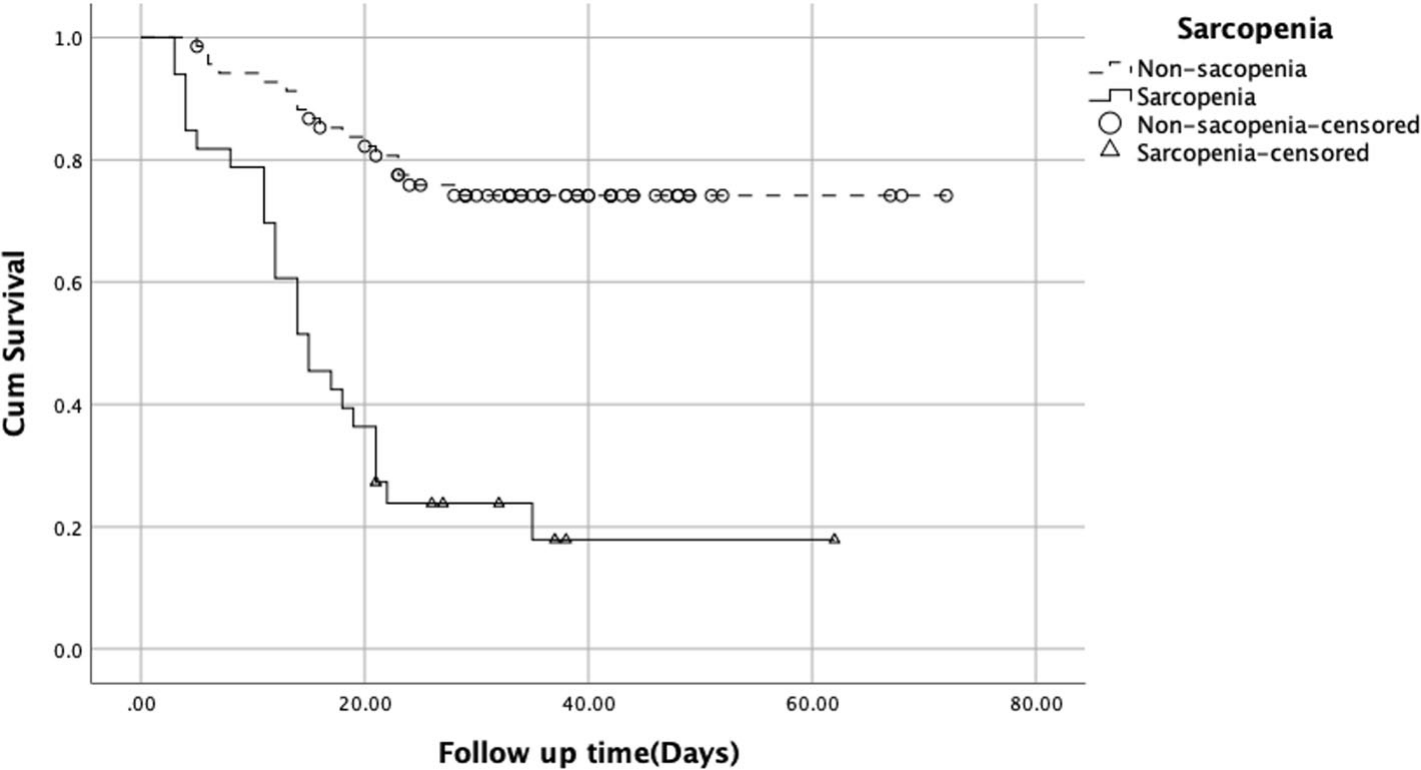
Kaplan-Meier estimates of the rate of severe disease among older COVID-19 patients with sarcopenia and non-sarcopenia (Log Rank test, *p* < 0.001))

We presented the survival curves of two group regarding the severe disease in Fig. 1. There was significant difference between high and low risk of sarcopenia group (Log Rank test, *p* < 0.001).

In the Cox proportional hazards model, the unadjusted hazard ratio of severe disease was 5.27 (95%CI, 2.83–9.82) for patients with a high risk of sarcopenia than those without (Table 3). The association persisted after adjusting for age, fatigue, dyspnea, anorexia, hemoglobin, neutrophil, lymphocyte, D-dimer, procalcitonin, BNP (Brain Natriuretic Peptide) and T cell subsets (CD3+, CD4+, CD8+). The hazard of having severe disease among patients with a high risk of sarcopenia was almost as 3-fold as those without (hazard ratio = 2.87, 95% CI: 1.33, 6.16).

**Table 3:** Association between sarcopenia risk and severe disease

	Unadjusted model	Adjusted model
Sarcopenia risk	Hazard ratio (95% confidence interval)	
Low risk	Ref.	Ref.
High risk	5.268(2.828–9.816)	2.867(1.334–6.161)

Adjusting for age, fatigue, dyspnea, anorexia, hemoglobin, neutrophil, lymphocyte, D-dimer, procalcitonin, BNP and T cell subsets (CD3+, CD4+, CD8+)

*Abbreviations*: *BNP* Brain Natriuretic Peptide

## Discussion

In the present study, we focused on a cohort of 114 senior patients (age ≥ 60) tested positive for COVID-19 in order to examine the association between a high risk of sarcopenia and progression to severe case among older COVID-19 patients. The study showed that higher risk of sarcopenia, assessed by a simple screening tool, was an independent risk factor of the progression to severe diseases among older COVID-19 patients. These results suggested that a quick and inexpensive test for sarcopenia risk could help timely identify high-risk patients, which in turn accelerate the timeline for necessary treatments.

Results from our study showed that COVID-19 patients with a high risk of sarcopenia had higher frequency of dyspnea, and this might be related to the decreased muscle strength in the respiratory system caused by sarcopenia. Previous studies made meaningful findings regarding the association between sarcopenia and respiratory diseases or complications. A study showed that patients who were entered into the Intensive Care Unit (ICU) due to the need for mechanical ventilation had a more positive correlation between respiratory insufficiency and sarcopenia [29]. Another study found that sarcopenia and pneumonia were closely related, which supports our conclusion [30]. Moreover, a study from Peru showed a higher incidence rate of community-acquired pneumonia among subjects diagnosed with sarcopenia [31].

After adjusting for the potential effects of dyspnea, a high risk of sarcopenia was still significantly associated with the progression of patients’ conditions. One possible explanation for this is that patients’ worsened conditions may be due to the presence of sarcopenia, which leads to a decline in the compensatory function of respiratory muscles in these patients when suffering from COVID-19, causing the clinical manifestations of decompensation to appear earlier and more acute. Evidence from a previous study can support this conjecture [32]. Said cohort study was conducted to investigate the association between muscle mass and outcome of pneumonia patients. The results showed that lower skeletal muscle mass was associated with higher 90-day mortality rate among patients with pneumonia. Some other studies concluded that protein intake and physical excise could maintain or improve muscle mass and function, which were believed to accelerate recovery and improve prognosis of older patients [33–35].

Above all, although there are many studies showing that age is closely related to the prognosis of patients with COVID-19 [36, 37], after adjusting for age, a high risk of sarcopenia is still an independent risk factor for the older in this study. And this result is consistent with our research hypothesis, that is, it is not age itself, but sarcopenia which contributed to the aggravation of patients’ COVID-19 condition. To investigate the mechanism of this phenomenon, studies have also shown that in addition to the decline in respiratory muscle function, inflammation and immune dysfunction are also closely related to sarcopenia [38–40].

Recent studies suggested that inflammatory storm is an important factor in the progression of COVID-19 patients’ prognosis [41, 42]. The presence of chronic inflammation can accelerate muscle loss and degradation, while muscle recovery can reduce the impact of chronic inflammation on the body to a certain extent [43]. IL-6, which is related to the pathogenesis of sarcopenia [44], plays an important role in the inflammatory storm of COVID-19 [45, 46]. In addition to activating immune system function against pathogens, IL-6 signaling is also a part of age-related chronic inflammation [47] and is related to the pathogenesis of sarcopenia [40]. Chronic low-grade inflammation is associated with prolonged exposure to IL-6 signaling [48], which may contribute to the progression of COVID-19 in older patients [49, 50]. In addition, the dynamic balance of skeletal muscle could contribute to the maintenance of healthy immune function [47].

Lastly, this study still has room for improvement which can be achieved through addressing several limitations. First of all, this study was conducted in a single isolated hospital with limited sample size. A multi-site large-scale cohort study of COVID-19 patients from all over the world will help further investigate the association between sarcopenia and COVID-19 prognosis. Secondly, this study measured sarcopenia risk by the SARC-F scale due to restrictions brought on by the epidemic-preventive conditions. Although SARC-F is a screening tool for sarcopenia, not a diagnostic instrument, partly due to its debatable sensitivity, it has well repeatability and high specificity with good screening effect. In addition, as a brief scale, it can be easily performed in a busy clinic setting and could minimize the exposure risk to COVID-19 infection among healthcare providers [51–53]. Future studies should adopt more accurate muscle quantification tests and a combination of multiple diagnostic methods to assess sarcopenia. Finally, our observation time was too short to observe the patient’s final outcome and long-term prognosis.

## Conclusion

This study found that patients with a higher risk of sarcopenia have a greater risk of developing severe COVID-19 symptoms, and that using a simple and feasible scale can help us timely identify high-risk patients. We should never give up older patients prematurely during a pandemic. Instead, we need to find the cause of their exacerbation or even death, intervene in advance, and save lives.

## Data Availability

The datasets used and/or analyzed during the current study are available from the corresponding author on reasonable request. Dr. Jirong Yue and Dr. Yan Kang had full access to all the data in the study and take responsibility for the integrity of the data and the accuracy of the data analysis.

## Abbreviations

COVID-19: Coronavirus disease 2019
ICU: Intensive care unit
WHO: World health organization
CRP: C-reactive protein
CD4+: Cluster of differentiation 4
CD8+: Cluster of differentiation 8
